# Ethiopian Health Extension Workers' Training Status and Perceived Competency

**DOI:** 10.4314/ejhs.v33i1.6S

**Published:** 2023-04

**Authors:** Mekdes Demissie, Netsanet Abera, Leake Gebremeskel, Daniel Tadesse, Tegene Legese Dadi, Tadele Zebere, Setegn Tigabu, Fasil Walelign Fentaye, Yibeltal Kiflie Alemayehu, Alula M Teklu, Gimay Medhin, Seleshi Zeleke

**Affiliations:** 1 College of Health and Medical Sciences, Haramaya University, Ethiopia; 2 Centre for Innovative Drug Development and Therapeutic Studies for Africa (CDT-Africa), College of Health Science, Addis Ababa University; 3 College of Medicine and Health Sciences, Hawassa University, Ethiopia; 4 College of Medicine and Health Sciences, Aksum University, Ethiopia; 5 MERQ Consultancy PLC, Addis Ababa, Ethiopia; 6 College of Social Sciences and Humanities, Hawassa University, Ethiopia; 7 Wollo University, School of public health; 8 Department of Health Policy and Behavioral Sciences, School of Public Health and Tropical Medicine, Tulane University, New Orleans, USA; 9 Department of Health Policy and Management; Jimma University, Ethiopia; 10 Aklilu Lemma Institute of Pathobiology, Addis Ababa University, Ethiopia; 11 School of Psychology, Addis Ababa University, Ethiopia

**Keywords:** Pre-service training, primary care, health extension workers, community health workers, low-income country, Ethiopia

## Abstract

**Background:**

Training and work experience are critical inputs for delivering quality health services. However, no nationwide assessment has been conducted on the status of training and the competency of Health Extension Workers (HEWs). Therefore, this study aimed to assess HEWs' pre-service training status and perceived competency in Ethiopia.

**Methods:**

The study was conducted in all regions and all HEWs training institutions in Ethiopia. We used cross-sectional study design with a mixed method approach that included 585 HEWs, 1,245 HEW trainees, 192 instructors, and 43 key informants. Descriptive statistics and thematic analysis were used to analyse quantitative and quantitative data respectively.

**Result:**

Twenty-six percent of the HEWs said that they were competent to deliver all the HEP activities, and 73% of the HEWs said that they could confidently deliver 75% of the HEP activities. Receiving in-service training and having level III/IV qualifications are positively associated with the competency of HEWs. Similarly, HEP trainees perceived themselves as highly competent in executing their professional work, except in using computer and mobile health technology. Both instructors and trainees rated the quality of the curriculum and course materials positively. However, basic services and facilities in most training institutions were perceived to be inadequate. Additionally, individual learning, problem-solving, case-analysis, and assessment methods such as project work and portfolios were rarely practiced.

**Conclusions:**

Although the perceived competence of HEW trainees is high, the HEWs' training is not provided as per the curriculum because of limited resources. All the necessary resources should be made available to produce competent HEWs.

## Introduction

During the early 1990s, communicable diseases, nutritional deficiencies, and maternal and child illnesses were the major public health problems and causes of mortality in Ethiopia (1). In 2003, the Ethiopian government launched the Health Extension Program (HEP) to improve access to basic health services (2-4). Health extension workers (HEWs) are the key actors for implementing the HEP (5), and the provision of quality HEP services depends on the competency of HEWs. Competency is a state of being capable and having the required knowledge and skills (6), adhering to implementation guidelines, and having the capacity to mobilize a community (7,8). Such competency is acquired through pre- and in-service training, and work experience (9).

According to the HEP guideline, HEWs should have one year of pre-service training (10), which is a relatively longer duration compared to the training given to community health workers in other low- and middle-income countries (LMICs) (11). Besides, integrated refresher training that covers key priority topics of HEP is designed as in-service training to improve the competency of HEWs (12). This training plays a key role in building the competency of HEWs (6,9,13). Moreover, supportive supervision and technical support (14) and longer years of service (15) are important inputs in producing competent professionals.

Studies in Ethiopia have shown inconsistencies in HEWs' competency in delivering various HEP packages. For example, HEWs were found competent in the provision of long-acting family planning methods and management of childhood illnesses (16), but they were found to lack competency in the provision of antenatal and delivery care services and managing complications in pregnancy (2), which is partly due to a lack of skills (17,18). Although all these evidences are very useful, similar information generated from national survey has more potential of influencing relevant HEP related policies. The national HEP assessment was conducted for the first time from October 2018 to October 2019 with the general objective of understanding the status of the HEP in Ethiopia (19). The aim of this paper is to report the perceived competency and pre-service training status of HEWs using data extracted from the national HEP assessment.

## Materials and Methods

**Context**: The HEP assessment included all regions and two city administrations (Addis Ababa and Dire Dawa). Each region is divided into woredas, which are further divided into kebeles (sub-districts). There were 23 national, government-run HEW training institutions during the study period (19). The HEP enrolment criteria were willingness to work in the community in which they are recruited, knowledge of the local language, being at least 18 years of age, having completed 10^th^ grade, and being female. In rural areas, the education-based criterion was reduced to having completed the 4^th^ grade, and men are also eligible (20). HEWs are salaried by the government to provide HEP services at health posts (HP) and through home-to-home visits and outreach services. According to the HEP, each kebele was expected to have an HP staffed with at least two HEWs. HEWs were required to take a certificate of competency (COC) test, which was mandatory for their career development and acceptance for further education.

**Study design**: This study was conducted as part of the national survey that evaluated the Ethiopian HEP in 2019. The study was cross-sectional with mixed method approach, with participants including (i) working HEWs, (ii) HEP trainees, (iii) professionals in various leadership positions and instructors working in HEWs teaching institutions, and (iv) COC assessors, and focal persons.

### Sample Size and Sampling Techniques

**Quantitative study-sample size and sampling**: The HEW competency study was embedded within a national HEP assessment in which several objectives were addressed. The sample size was calculated to estimate the percentage of HPs with at least one HEW trained to diagnose and treat malaria, which resulted in 372 HPs (19). This required covering 64 woredas. Multistage sampling was used to identify study woredas from nine regions, HPs within each woreda, and a maximum of two HEWs per HP, which resulted in 585 HEWs from 372 HPs. In the two city administrations, HEP is implemented by nurses who hold diplomas in nursing; these health care providers were not included in this paper.

In the 2018/2019 academic year, 11,500 trainees were enrolled in HEW programs in Ethiopia. A minimum sample size of 1,600 was estimated, with the assumption that at least 50% of the trainees would have a high level of perceived competency, a 2.5% margin of error, 95% confidence in the estimate, application of a finite population correction factor, a 10% non-response rate, and a 10% increase to allow subgroup analysis. Two of the 23 training institutions were not included in the survey. One training institution in Hawassa closed before schedule for unknown reasons; hence, students and instructors were not accessible for data collection. Data collection in the Pawe Health Science training institution was impossible for security reason. The total sample size of trainees was divided among the training institutions. The response rate of 77.8% resulted in a total sample size of 1,245. Every instructor was approached for the quantitative survey because their total number was manageable.

### Qualitative Study-Sample Size

**Trainees and instructors**: We interviewed 43 participants: three deans, seven department heads, seven instructors, nine trainees, seven internship supervisors, six COC assessors, and four focal persons. We used purposive sampling to select all participants except trainees. The COC assessors and focal persons were from regional health bureaus (RHBs), regional and federal technical and vocational education and training (TVET) agencies, and the Ministry of Health (MoH).

**Outcome and selected independent variables**: The definition of outcomes, selected independent variables, response categories of the variables, and internal consistency measures are summarized in Table 1. We used five domains to measure the competency of HEWs: maternal and child health (MCH), family planning (FP), disease prevention and control, hygiene and sanitation, and health education and communication. To measure the competency of HEP trainees, we used a single domain consisting of 21 items. In addition, we assessed training institutions' facility and infrastructure-using an observation document review checklist.

**Table 1:** Description of key variables and internal consistency measures of composite variables

Measured outcome	Number of items in the tool	Possible range in total score	Internal consistency (Cronbach's alpha)
**HEP trainees' questionnaires**			
**Trainees' perceived competency**	21	21–105	0.94
Satisfaction with the course	6	0–30	0.81
Trainees' perception of HEP	10	10–50	0.89
Satisfaction with teaching–learning process	4	0–16	0.81
Self-esteem about capability	5	5–20	0.80
Trainees' perception of instructors' competency	13	0–26	0.91
Satisfaction with institutions' facilities	14	14–70	0.84
**Instructors' questionnaires**			
Perception of relevance of HEP	10	10–50	0.86
Satisfaction with infrastructure and services	14	14–70	0.87
Perception of quality of trainees	13	13–65	0.89
Methods of instruction	10	0–40	0.72
Assessment methods	7	0–35	0.7
Satisfaction with teaching–learning process	4	0–16	0.87
Perception of trainees' competency	17	17–85	0.95
**HEWs' questionnaires**			
**HEWs' perceived competency**			
MCH	12	0–36	0.89
FP	4	0–12	0.79
Disease prevention and control	3	0–9	0.74
Hygiene and sanitation	4	0–12	0.90
Health education and communication	12	0–36	0.94
**In-service training**			
**MCH (ICCM)**	1	0–1	
Disease prevention and control	5	0–5	
**Knowledge of HEWs**			
Knowledge of FP	5	0–5	0.67
Knowledge of immunization	9	0–9	0.68
**HEWs' supervision**	7	7–28	0.81
**Satisfaction of HEWs**	33	33–132	0.95

**Data collection methods and quality assurance**: We developed and used structured questionnaires, a semi-structured interview guide, and an observation checklist. The quantitative questionnaire and qualitative topic guide were used to assess the relevance of the HEW training curricula, the quality of the course materials, delivery, student assessment methods, and the HEP trainees' perceived competency for the courses they completed and HEWs' competency. The observation checklist was used to assess the availability and adequacy of human and material resources. The tools were translated from English into four local languages (Amharic, Afan Oromo, Tigrigna and Nuer) and pretested before the field data collection. Data collectors and supervisors were trained for 10 days. All qualitative interviews were audio-recorded.

**Statistical analysis**: We used descriptive statistical methods to summarize the quantitative characteristics of study the participants and the readiness of training institutions. We used a t-test to compare the agreement of instructors and trainees on the relevance and appropriateness of the HEP training curriculum. Cornbrash's alpha was used as a measure of internal consistency of items included in various measures, where any value more than 0.7 indicates acceptable internal consistency of the items. As measure of degree of association, we used crude and adjusted incidence rate ratio (IRR), with corresponding 95% confidence intervals generated from Poisson regression and negative binomial regression. In managing qualitative data, first, all interviews were translated into English and coded using NVivo 12. The codes were categorized into sub-themes and then themes, partly based on the topic guide. Finally, all the quantitative and qualitative findings were interpreted together.

**Ethical considerations**: The conduct of HEP assessment was approved by the Institutional Review Board of the Ethiopian Institute of Public Health (EPHI-IRB-151-2018). Verbal informed consent was obtained from all study participants.

## Results

**Characteristics of study participants**: We interviewed 585 HEWs, 1,245 HEP trainees, and 192 instructors. The mean (SD) age of HEWs and trainees was 26.4 (4.8) and 23.2 (4.5), respectively. More than 90% of the HEWs were females and held Level III/IV qualifications, and more than 50% were COC certified. Among the trainees, 45% were upgrading to Level III/IV. The mean (SD) age of instructors was 30.5 (5.8) years, 78% were males, and more than two thirds were first-degree holders (Table 2).

**Table 2:** Socio-demographic characteristics of the participants

Variable	HEW (N = 585) Number (%)	Trainees (N = 1245) Number (%)	Instructors (N = 192) Number (%)
Age in years: mean (SD)	26.4 (4.8)	23.2 (4.5)	30.7 (5.8)
Sex			
Female		1,190 (95.5)	43 (22.4)
Male		55 (4.5)	149 (77.6)
Marital status			
Single	159 (27.2)	666 (53.4)	
Married	400 (68.4)	568 (45.6)	
Divorced/separated/widowed	26 (4.4)	11 (0.9)	
Service year in year: mean (SD)	6.9 (4.4)		4.6 (3.9)
Level of HEW training			
I/II	51 (8.7)		
III	294 (50.3)		
IV	240 (41.0)		
COC certification			
COC certified	333 (56.9)		
Not certified	252 (43.1)		
Grade level attained			
Grade 4–8		15 (1.2)	
Grade 10		964 (77.4)	
Grade 11–12		266 (21.4)	
Program attending			
New HEP trainees		653 (52.5)	
Upgrading to Level III/IV		562 (45.1)	
Urban HEP		30 (2.4)	
Instructors' educational qualifications			
BSc level professionals			130 (67.7)
Masters in public health			31 (16.2)
Masters in clinical in nurse/midwife			21 (10.9)
Diploma			10 (5.2)
Instructors' fields of study			
Nursing			80 (41.7)
Public health			64 (33.3)
Midwifery			31 (16.2)
Environmental health			14 (7.3)
Lab technician/technologist			1 (0.5)
HEW			2 (1.0)

**Training institutions' readiness to provide pre-service training-Relevance of the program**: Instructors (n = 192; mean = 21.78; 95% CI: 20.8, 22.7) and trainees (n = 1245, mean = 20.54, 95% CI: 20.33, 20.76) positively rated the quality of the curriculum and course materials. They mentioned that the curriculum has clear objectives with proper depth and breadth for its intended purpose. The mean score of the perceived relevance of the HEP curriculum was relatively higher among instructors than trainees (mean difference = 1.24, 95% CI: 0.42, 2.05). Participants in the qualitative study reported that although the HEP curriculum is well-designed and relevant, the time allotted to cover the contents is inadequate and needs revision (Table 3).

**Table 3:** Examples of quotations for the qualitative finding

Themes		Quotations
Relevance of the program	Relevance of HEP curriculum	“The sixteen packages included in [the] HEP curriculum are directly related to community major health problems, community-focused, and problem solvers… In general, the program is vital to our communities.” (In-depth interview with Head of Department, Gambella)

	*Time allotted to cover topic is perceived to be inadequate*	“The training program is extensive. The HEP content and the amount of time allotted to cover it do not match. A number of courses are required to be covered within a short period of time, which will overburden the trainees.” (KII, Head of the HEP Department, Regional State)

Teaching and assessment methods	Use of lecture method rather than practical teaching method	“…the instructors who don't know well about the packages and HEP, teach HEP trainees like nurses and midwives mostly using lecture method, no technical and vocational and education training [TVET] philosophy is being implemented.” (KII, COC Assessor, Health Science Training Institution) As a part of the TVET system, HEP training is expected to be 70% practical and 30% theoretical. In reality, this is not the case. Most teachers spend most of the time provided for practical training in class. As a result, students have limited opportunity to gain practical experience.

Factors affecting HEWs' or trainees' low competency	Some trainees have limited capacity for learning	“The main problem is the poor academic background of trainees… Their competence problem is, therefore, directly related to the selection of incompetent trainees during their entrance.” (KII, Instructor, Oromia Region) “I think the main problem occurred during the selection and screening of HEWs for training. They are not carefully recruited, and attention is not paid for the selection process.” (KII, Woreda Health office, Process Owner) “The HEP included those who are left at home due to poor academic performance or those who failed Grade 10 or 12 and were unable to attend college.” (KII, SNNPR RHB)
	
	Low capacity in understanding the language of instruction (English)	“Not all HEWs are able to fully understand the instructional language of the training. The language barrier is a great challenge to HEWs. Some of the HEWs want the training modules to be translated into their local languages. This is one of the main challenges in providing in-service training.” (KII, RHB Coordinator) “The selection criteria were not as strong as selection criteria used for diploma programs. I think that is why HEWs have trouble passing the COC exams and improving their education. As I mentioned earlier, we may need to modify the recruitment criteria to get competent candidates.” (KII, Amhara RHB)

**Adequacy of input in the training institutions**: Most trainees reported that the basic services and facilities necessary for the training are available, but more than 40% of them mentioned that they were inadequate. Similarly, about two thirds of instructors reported low or moderate satisfaction with the available facilities. One third of the trainees reported having no internet access, and 75% of the instructors reported low to moderate levels of satisfaction with internet access despite its availability (Supplementary File 2). In addition to inadequacy of inputs, the qualitative study participants mentioned that very few or no staff are exclusively dedicated to the HEP department. Hence, instructors from different departments are assigned to teach HEP trainees. **Teaching and assessment methods**: Instructors mentioned that they frequently used lectures (82.8%), demonstrations (65.6%), and practical training (59.4%) as their major teaching methods. Seminars, individual learning, problem-solving, and case analysis were used either sometimes or rarely. Written exams, assignments, practical exams, and observation (63%) were the most used assessment methods. Project work and portfolio assessment were less frequently used (Table 4). Similarly, participants in the qualitative study mentioned the lecture method as the most used in the TVET system, where it is recommended that 70% of training time should be used for practical sessions (Table 4).

**Table 4:** Trainees' and instructors' perceptions of input in the teaching institution

Trainees' perceptions	Not available	Available but inadequate	Available and Adequate
Classroom facility	19 (1.5)	558 (44.8)	668 (53.7)
Library facility	58 (4.7)	538 (43.2)	649 (52.1)
Computer services	1 (0.1)	691 (76.5)	211 (23.4)
Internet services	462 (37.1)	597 (48.0)	186 (14.9)
Women's toilets	24 (1.9)	693 (55.7)	528 (42.4)
Men's toilets	24 (1.9)	659 (52.9)	562 (45.1)
Water services	143 (11.5)	500 (40.2)	602 (48.3)
Demonstration room	41 (3.9)	713 (57.4)	491 (39.4)
Skills lab	247 (19.9)	629 (50.5)	369 (29.6)
Lab equipment	233 (18.7)	655 (52.6)	357 (28.7)
Practice site	56 (4.5)	446 (35.8)	743 (59.7)

**Instructors' perceptions**	**Low**	**Moderate**	**High**

Library services	41 (21.4)	63 (32.8)	88 (45.8)
Availability of books in your field of study	51 (29.5)	57 (29.7)	84 (43.8)
Multiple copies of core books in your field of study	51 (26.1)	66 (34.4)	75 (39.1)
Library loans	62 (32.3)	55 (28.6)	75 (39.0
Instructors' offices and office materials	81 (42.2)	57 (29.7)	54 (28.1)
Equipment and materials for practical courses	59 (30.7)	63 (32.8)	70 (36.4)
Personal computers	59 (30.7)	34 (17.7)	99 (51.5)
Computer lab	70 (32.5)	45 (23.4)	77 (40.1)
Internet services	88 (45.8)	67 (34.9)	37(19.3)
Latrines	54 (28.1)	60 (31.3)	78 (40.6)
Water services	61 (31.8)	56 (29.2)	75 (39.0)
Electricity	34 (17.7)	43 (22.4)	115 (59.9)
Skills labs	50 (26.0)	62 (32.3)	80 (41.6)

### Perceived Competency of Trainees and HEWs

**Perceived competency of HEP trainees**: Most of the trainees perceived themselves as highly competent in executing their professional duties. They considered themselves incompetent only in using computer and mobile health technology. However, instructors had concerns about trainees' theoretical and practical competency and intellectual maturity. Additionally, they evaluated trainees' attitudes toward HEP and their communication skills as moderate or poor. In contrast, the instructors positively evaluated trainees' motivation to learn and trainees' discipline, with special focus on interest in the course, class attendance, social maturity, and punctuality. The details are presented in table-5.

**Table 5:** Frequency of teaching methods and assessment techniques employed in training HEWs

	Never/rarely N (%)	Sometimes N (%)	Mostly/always N (%)
**Teaching method**			
Lecture	7 (3.6)	26 (13.5)	159 (82.8)
Demonstration	16 (8.3)	50 (26.0)	126 (65.6)
Practical training/guided practice	24 (12.5)	54 (28.1)	114 (59.4)
Group discussion	15 (7.8)	103 (53.7)	74 (38.5)
Individual learning	95 (49.5)	73 (38.0)	24 (12.5)
Problem solving	68 (35.4)	74 (38.5)	50 (26.0)
Role play	46 (23.9)	93 (48.4)	53 (27.6)
Seminar	113 (58.8)	59 (30.7)	20 (10.4)
Case analysis	93 (48.4)	64 (33.3)	35 (18.2)

**Assessment method**			

Written exam/test	5 (2.6)	10 (5.2)	177(92.2)
Assignment	6 (3.1)	58 (30.2)	128 (66.7)
Questioning	15 (7.8)	48 (25.0)	129 (67.2)
Practical exam	21 (10.9)	55 (28.7)	116 (60.4)
Observation	24 (12.5)	47 (24.5)	121 (63.0)
Project work	69 (35.9)	79 (41.2)	44 (22.9)
Portfolio assessment	92 (47.9)	42 (21.9)	58 (30.2)

The trainees' perceived mean (SD) competency was 52.8% (8.5) in the instrument measure ranges from 0 to 63. In the fully adjusted model, trainees' perceived competency was positively associated with their satisfaction with the course and the teaching–learning process, being female, being married, having completed Grade 10 or higher, being older, and being in the process of upgrading to Level IV (Supplementary File 5). In the qualitative study, participants explained that those who held Level IV were academically more competent than those who held Level III for two reasons: Level III trainees (1) are recruited from Grade 10 or 12 without prior exposure to the HEP and (2) usually join the HEP after being classified as unfit to join other technical schools. Participants also mentioned that Level III trainees were better in practical skills than in theoretical knowledge because their English-language proficiency was a barrier to reading and understanding modules prepared in English.

**Perceived competency of HEWs**: Among 585 HEWs, 73% reported that they can do 75% of HEP activities that were listed to assess their overall perceived competency. The total score of this competency scale ranges from 0 to 81, and the perceived competency mean (SD) was 61.3 (15.5). HEWs who had in-service training in MCH and disease prevention and control were more competent than those who had no training. Similarly, being a Level III or IV HEW was positively associated with better perceived competency compared to Levels I and II (Table 6).

**Table 6:** Perceived competency of trainees and associated factors

Trainees' competency	IRR (95 % CI)	95 % CI
Trainees' perception about HEP*	0.99	0.99, 1.01
Trainees' self-esteem about their own capability*	1.01	1.00, 1.01
Training institution service availability*	0.99	0.98, 0.99
Trainees' satisfaction with course provided*	1.00	0.99, 1.00
Trainees' satisfaction with teaching–learning process*	1.00	0.99, 1.00
Age*	1.00	1.00, 1.00
Training level	1.06	1.04, 1.09
New HEP trainees		
Upgrading to Level IV	1.08	1.05, 1.11
Urban HEP	1.07	1.00, 1.14
Gender		
Male		
Female	1.06	1.00, 1.10
Marital Status		
Single		
Married	1.03	1.00, 1.05
Widowed	0.97	0.88, 1.06
Residence		
Urban		
Rural	1.01	0.99, 1.03
Completed grade		
Grade 4–8		
Grade 10	1.10	1.01, 1.20
Grade 11–12	1.10	1.02, 1.19

* Continuous variable

Regarding perceived competency in a specific HEP service package (table 7), 73% of HEWs reported that they can do 75% of the MCH activities by themselves. In reference to the maximum score of 36, the mean perceived MCH competency score was 21.3 (SD = 6.0). Regarding perceived competency in FP services, 61% of the HEWs stated that they could provide all FP-related services without support, whereas 40% reported that they could provide three out of four services. In reference to the maximum score of 12, the HEWs' mean perceived competency score in delivering FP was 7.6 (SD = 2.4).

**Table 7:** Associations of perceived competency of HEWs in five HEP packages and various background characteristics

Predictors	MCH competency	FP competency	Disease prevention competency	Hygiene competency	Health education and communication competency
Have in-service training					
MCH training taken	1.10 (1.05, 1.15)	1.18 (1.09,1.29)			
Disease prevention and control			1.10 (1.00, 1.20)		
Hygiene and sanitation				0.95 (0.62, 1.45)	
Knowledge of HEWs					
Knowledge on FP	1.03 (1.02, 1.04)	1.01 (0.98, 1.04)			
Knowledge on immunization	1.03 (1.00, 1.04)				
Service year	1.01 (1.01, 1.02)	1.02 (1.01, 1.04)	1.01 (0.99, 1.03)	0.62 (1.00, 1.04)	. 1.03 (1.02, 1.04)
Level of HEW training					
III	1.23 (1.13, 1.33)	1.15 (1.01, 1.32)	1.16 (0.98, 1.36)	1.05 (0.88,1.25)	1.15 (1.04, 1.27)
IV	1.26 (1.15, 1.37)	1.17 (1.02, 1.03)	1.21 (1.03, 1.42)	1.03 (0.86, 1.23)	1.14 (1.03, 1.26)
COC certification					
COC certified	1.06 (1.01, 1.10)	1.10 (1.03, 1.19)	1.02 (0.94, 1.11	1.06 (0.96, 1.16)	1.06 (1.00, 1.12)
Supervision	1.00 (0.99, 1.00)	1.00 (0.99, 1.01)	1.00 (0.99, 1.01)	1.00 (0.98, 1.01)	1.00 (0.99, 1.00)
Satisfaction	1.00 (1.00, 1.01)	1.00 (1.00, 1.00)	1.00 (0.99, 1.01)	1.00 (0.99, 1.00)	1.00 (0.99, 1.00)
HEWs who were learning as part time during data collection					
Yes – Health fields	1.11 (0.99, 1.24	1.01 (0.84, 1.22)	1.12 (0.90, 1.39)	0.91 (0.69, 1.20)	0.99 (0.85, 1.15
No – Started education	1.09 (1.03,1.15)	1.04 (0.95, 1.13)	1.01 (0.90, 1.12)	1.03 (0.91, 1.17)	1.03 (0.96, 1.11)
HEW residence					
Within kebele	1.00 (0.95, 1.04)	0.98 (0.91, 1.05)	1.03 (0.94, 1.11)	0.97 (0.88, 1.07)	0.98 (0.93, 1.04)
HEW age	1.00 (0.99, 1.00)	0.98 (0.98, 0.99)	0.99 (0.98, 1.00)	0.99 (0.98, 1.00)	0.98 (0.97, 0.99)
Marital status					
Never married	0.93 (0.87, 0.99)	0.98 (0.88, 1.10)	1.00 (0.88, 1.14)	0.93 (0.80, 1.09)	0.95 (0.87, 1.04)
Separated/divorced	1.00 (0.91, 1.10)	1.04 (0.89, 1.21)	1.05 (0.88, 1.26)	0.99 (0.79, 1.23)	1.04 (0.92, 1.18)
Number of children					
1–2 children	1.03 (0.97, 1.10)	1.07 (0.96, 1.20)	1.03 (0.90, 1.18)	0.99 (0.85, 1.15)	1.02 (0.94, 1.11)
>=3 children	1.05 (0.97, 1.14)	1.07 (0.93, 1.22)	0.99 (0.85, 1.17)	1.03 (0.85, 1.23)	1.02 (0.92, 1.14)

Figures reported in the body of the table are adjusted incidence rate ratio (IRR) and 95% confidence interval for the IRR (95 % CI).

In the table, Not taking in-service training, Level I/II HEW training, Not COC-certified; HEWs who were learning in non-health-related fields, living outside working kebele, currently married, not having child are reference categories.

FP = family planning; MCH = maternal and child health

In disease prevention and control, 82% of the 585 HEWs reported that they could perform disease prevention and control activities by themselves. In reference to the maximum score of 9, the HEWs' mean perceived competency in delivering disease prevention and control was 5.5 (SD = 1.8). Additionally, 89% of the HEWs reported that they could perform all HEP activities related to hygiene and environmental sanitation without support. Regarding the delivery of health education and communication, in reference to the maximum score of 36, the HEWs' mean perceived competency was 22.8 (SD = 6.2). About half (49%) reported that they could accomplish all activities without support.

The adjusted effect of different covariates on the competency of HEWs in the five main packages is summarized in Table 3. HEWs who had received in-service training in MCH were more competent in delivering MCH-related services than those who had not received in-service training. Additionally, a one-point increase in the knowledge score of HEWs on FP and immunization was associated with a 3% increase (adjusted rate ratio: 1.03; 95% CI: 1.02, 1.04) in the rate ratio of their MCH care competency. Additionally, HEWs holding Level III or IV and being COC certified were associated with significantly higher competency in delivering MCH and FP services compared to Level I or II HEWs and noncertified HEWs, respectively.

## Discussion

To the best of our knowledge, this is the first national assessment conducted to investigate the readiness of HEW training institutions and HEWs' perceived competency. In this study, the HEP curriculum was perceived as relevant, but the basic facilities necessary for training and practical teaching were inadequate. Instructors and trainees reported the trainees' competency differently. HEP trainees perceived themselves as highly competent, whereas instructors perceived them to have low to moderate levels of competency. In-service training, better knowledge about and skills in FP and immunization, and being Level III or IV HEWs are associated with higher levels of perceived competency.

HEP training was designed to have 70% practical and 30% theoretical sessions (21,22). However, less than one third of the training institutions have adequate facilities for practice sessions, and only 60% have practice sites. This indicates that training institutions need to be supplied with the necessary inputs to produce HEWs with the required knowledge and skills (23). Similar challenges were observed about 15 years ago, with the exception of improvements seen in classrooms and libraries (24). This is an indication that little effort has been made in equipping training institutions. Similarly, despite few or no staff being exclusively assigned to the HEP department, the number, qualifications, and specializations of instructors in training institutions have improved since the previous study (25).

Although studies have shown the effects of teaching methods, tools, and materials on the quality of education and training (26,27), our findings show that some teaching methods, such as problem-solving, seminars, and case analysis, are rarely used. Inadequate resources such as internet access and well-equipped libraries were mentioned as barriers and should be addressed as areas for intervention to improve the quality of pre-service training.

HEWs in the current study had low perceived competency, and only a quarter reported high perceived competency in delivering all HEP activity support, in line with previous studies (28). In Rwanda, the competency score of community health workers was more than 75% (29). Having sufficient clinical learning experience was also a significant predictor of competency for health professionals (30). The importance of training institution infrastructure to the competency of HEWs was further highlighted in a Nigerian study (31). These findings indicate that the skills and knowledge of trainees are highly dependent on the adequacy of inputs. HEWs with MCH-related in-service training in addition to pre-service training were more competent in MCH care compared to their counterparts. This indicates the key role of practice-based in-service training in enabling HEWs to acquire both the knowledge and the skills required and to improve their competency (10) and the service they provide for the community (32). Reports also showed that more in-service training was provided to HEWs in the area of MCH and FP by including task-shifting training in which HEWs are trained to provide long-acting reversible contraceptives (19,33).

In this study, Level III or IV certification more significantly associated with better competency to deliver all HEP packages than Level II certification. The difference in the learning outcomes of the two groups of HEWs might explain the difference in perceived competency. The lower-level HEWs are more likely focused on health promotion and health education (21,22), whereas the higher level HEWs (Levels III and IV) are more likely to have adequate skills and experience to perform promotive, preventive, and curative services. Being COC certified is also significantly associated with better MCH and FP competency.

One strength of the current study is having national coverage that included all regions and HEP teaching institutions. We also included different stakeholders and used a mixed-methods approach to generate evidence that is valid and generalizable across the country. However, the study is not without limitations. Two teaching institutions were not included, and the effects of this are unknown. Although competency was assessed with nonstandard tools, all the items were assessed by experts in the field and had acceptable internal consistency based on Cornbrash's alpha cut-off.

In conclusion, the HEP training was perceived as being well-designed and relevant. Nevertheless, most training institutions were found to have inadequate basic facilities and to focus on theoretical training. Additionally, the time allocated to cover the course content was perceived as inadequate. The findings reveal a need for capacity building of the training institutions to ensure that they have adequate resources for practical sessions, to improve the HEP admission criteria to recruit competent trainees, and to review the time allotted to cover the course. In-service training and upgrading HEWs to Level IV should be a continuous process to improve the competency of HEWs who are already on the job.
